# Pro-inflammatory and hyperinsulinaemic dietary patterns are associated with specific gut microbiome profiles: a TwinsUK cohort study

**DOI:** 10.1017/gmb.2024.14

**Published:** 2024-12-05

**Authors:** Ni Shi, Sushma Nepal, Rachel Hoobler, Cristina Menni, Mary C. Playdon, Daniel Spakowicz, Philippa M. Wells, Claire J. Steves, Steven K. Clinton, Fred K. Tabung

**Affiliations:** 1 Comprehensive Cancer Center, The Ohio State University, Columbus, OH, USA; 2 Interdisciplinary Ph.D. Program in Nutrition, The Ohio State University, Columbus, OH, USA; 3 Department of Nutrition and Integrative Physiology, College of Health, University of Utah, and Huntsman Cancer Institute, Salt Lake City, UT, USA; 4 The Department of Twin Research, Kings College London, London, UK; 5 Division of Medical Oncology, Department of Internal Medicine, College of Medicine, The Ohio State University, Columbus, OH, USA; 6 Division of Epidemiology, College of Public Health, The Ohio State University, Columbus, OH, USA

**Keywords:** Empirical Dietary Index for Hyperinsulinaemia (EDIH), Empirical Dietary Inflammatory Pattern (EDIP), insulin response, inflammation, microbiome, microbiota

## Abstract

Metabolic dietary patterns, including the Empirical Dietary Index for Hyperinsulinaemia (EDIH) and Empirical Dietary Inflammatory Pattern (EDIP), are known to impact multiple chronic diseases, but the role of the colonic microbiome in mediating such relationships is poorly understood. Among 1,610 adults with faecal 16S rRNA data in the TwinsUK cohort, we identified the microbiome profiles for EDIH and EDIP (from food frequency questionnaires) cross-sectionally using elastic net regression. We assessed the association of the dietary pattern-related microbiome profile scores with circulating biomarkers in multivariable-adjusted linear regression. In addition, we used PICRUSt2 to predict biological pathways associated with the enriched microbiome profiles, and further screened pathways for associations with the dietary scores in linear regression analyses. Microbiome profile scores developed with 32 (EDIH) and 15 (EDIP) genera were associated with higher insulin and homeostatic model assessment of insulin resistance. Six genera were associated with both dietary scores: *Ruminococcaceae_UCG-008, Lachnospiraceae_UCG-008, Defluviitaleaceae_UCG-011 Anaeroplasma*, inversely and *Negativibacillus, Streptococcus*, positively. Further, pathways in fatty acid biosynthesis, sugar acid degradation, and mevalonate metabolism were associated with insulinaemic and inflammatory diets. Dietary patterns that exert metabolic effects on insulin and inflammation may influence chronic disease risk by modulating gut microbial composition and function.

## Introduction

Gut microbial dysbiosis or imbalance has been associated with many diseases, including obesity, inflammatory bowel disease, cancer, neurodegeneration disorders, and others (Chen and Devaraj, 2018; Parekh *et al.*, 2015). Diet may impact health via its modulation of the gut microbiota, and the structure and function of the colonic microbiome may play a critical role in mediating dietary effects on health and disease (Pallister *et al*., 2017; Valdes *et al*., 2018; Zierer *et al*., 2018). In parallel with the rapid advances in understanding the role of host microbes in health and disease, there has been a growing appreciation of how dietary patterns, as opposed to individual components or nutrients, impact health. Our efforts have focused upon an empirical strategy to define novel dietary patterns that predict host blood biomarker concentrations that are strongly associated with disease risk (Shi *et al.*, 2021; Tabung *et al.*, 2016; Tabung *et al.*, 2016). Employing data from large prospective epidemiologic studies, we have defined novel dietary pattern indices based upon the ability to impact biomarkers of hyperinsulinaemia or chronic inflammation and designated as the Empirical Dietary Index for Hyperinsulinaemia (EDIH) and Empirical Dietary Inflammatory Pattern (EDIP), respectively (Shi *et al.*, 2021; Tabung *et al.*, 2016; Tabung *et al.*, 2016).

EDIH and EDIP have been examined in a series of epidemiological cohort studies with findings validating initial associations with risk and prognosis of multiple chronic metabolic diseases (Wang *et al.*, 2023) including obesity (Tabung *et al*., 2019), type 2 diabetes (Jin *et al.*, 2021; Lee *et al.*, 2020), cardiovascular disease (Li *et al.*, 2020), and several cancers (Aroke *et al*., 2020; Jin *et al*., 2023; Lee *et al*., 2019; Tabung *et al.*, 2018). A key gap in our knowledge regarding the mechanisms whereby EDIH and EDIP may impact health and disease risk concerns the complex interactions with the faecal microbiome. Changes in microbial structure and function in response to diet (Pallister *et al*., 2017; Zierer *et al*., 2018) may influence host metabolism and downstream functional effects, including modulation of inflammation and insulin resistance (Valdes *et al*., 2018).

This study employs a cohort with dietary assessment, serum biomarker assessment, and analysis of the faecal microbiome composition. Our study objectives were to identify the microbiota profiles associated with the EDIH and EDIP dietary patterns and assess their associations with circulating biomarkers of insulinaemia and inflammation. In addition, given that these dietary scores were being computed in a non-US population (TwinsUK cohort) for the first time, we conducted a construct validation of EDIH and EDIP dietary scores by evaluating their associations with circulating markers of insulin and inflammation. Quantifying how these variables are related can provide a basis for new hypotheses to inform dietary pattern intervention studies targeting the gut microbiome to reduce hyperinsulinaemia and chronic inflammation, to improve metabolic health, and reduce disease burden.

## Methods

### Study population

TwinsUK is a national adult twin registry in the United Kingdom initiated in 1992 and recruited more than 15,000 male and female community-dwelling twins aged 18–100 years, into the cohort (Spector and Williams, 2006). TwinsUK is a multidisciplinary platform providing deeply phenotyped and genotyped data for health- and social-related research with multiple visits and prospective follow-up (Verdi *et al*., 2019). After about 7,000 twins enrolled at baseline (1992–2004), more than half of them finished a follow-up visit between April 2004 and May 2007. From August 2007 to April 2012, the second wave of follow-up visits invited 3,125 women with at least one previous clinical visit. The third wave of follow-up visits was performed between May 2012 and May 2018, including 5,151 participants from the earlier waves. Since February 2019, a further wave of follow-up visits has been ongoing. Multiple questionnaires and clinical samples were collected during the baseline and follow-up visits.

The baseline food frequency questionnaire (FFQb) was collected between 1993 and 2001 from 4,472 participants. The FFQb collection included a maximum of three time points per individual and 5,414 records. The first wave of follow-up visits (follow-up 1) happened between 2004 and 2007, and no dietary data were collected. The second FFQ (FFQ2), corresponding to the second wave of follow-up visits (follow-up 2), data were collected in 2007 from 3,370 participants at only one time point. The third FFQ3 (follow-up 3) was from 2014 to 2018 among 5,440 participants, with two time points for some participants. Circulating biomarkers, clinical characteristics, and other longitudinal data were collected multiple times during the follow-up periods, including stool samples in the third wave of follow-up visits. In the current study, we conducted cross-sectional analyses linking dietary scores from FFQ3 with faecal microbiome data, as well as cross-sectional and longitudinal analyses examining diet and biomarkers at all three time points for data collection (Supplementary Figure 1). Ethical approval for the TwinsUK cohort study was obtained from St. Thomas’ Hospital Research Ethics Committee, and written informed consent was obtained from all study participants.

### Diet assessment in TwinsUK and calculation of EDIH and EDIP scores

In TwinsUK, habitual diet was estimated using a 131-item FFQ previously used in the European Prospective Investigation into Cancer and Nutrition study (Teucher *et al*., 2007). FFQ measurement characteristics have been evaluated and validated against urinary nitrogen, potassium, serum vitamin C, and carotenoids (Teucher *et al*., 2007). For participants with multiple clinic visits and FFQ data during one time point, we used the mean value as the final food intake value for each individual. The FFQ data estimated the frequency of intake of individual food items in servings per week. While most foods had the same portion size definition across the three FFQs, there were differences for a few food items, for example, the portion size for low-fat cottage cheese was medium serving in FFQb and tablespoons in FFQ2. Therefore, to improve the comparability of scores across the three FFQs and with external cohorts, we employed a standardised serving size strategy using the serving size information from the Nutrition Data System for Research (NDSR) software of the University of Minnesota (Schakel, 2001). NDSR is a dietary analysis program designed for foods (servings per day) and nutrient intake analyses. We used the 168 standardised serving-size food subgroups within the nine major food categories defined in the NDSR 2017 version. The serving size for each food subgroup was assigned based on the recommendations made by the 2000 Dietary Guidelines for Americans or Food and Drug Administration (FDA) serving sizes. We converted each food item in the TwinsUK FFQs into the NDSR serving sizes per day and assigned them to the appropriate NDSR food subgroups. We created two additional food groups for pizza and cream soup since they were disaggregated into ingredient levels in NDSR but treated as a whole food in the EDIH/EDIP score estimation process. We then calculated EDIH and EDIP scores based on the 170 standardised food subgroup servings and the components of each dietary pattern in TwinsUK, listed in Supplementary Table 1.

### Microbiome data assessment

During the third follow-up, faecal samples were collected for microbiome analysis (Verdi *et al.*, 2019). Faecal sample collection, bacterial DNA extraction, amplification, and sequencing have previously been described in detail (Bowyer *et al*., 2019; Goodrich *et al.*, 2016). Briefly, after 16 sRNA V4 variable regions were sequenced using the Illumina MiSeq platform, amplicon sequence variants (ASVs) were generated using the DADA2 pipeline (Callahan *et al*., 2016). Samples achieving a sequence depth of less than 10,000 were excluded. Taxonomy was assigned to the ASV sequences using the SILVA reference database, and reverse transcript sequences were included (Wells *et al*., 2020). Sequences that were unassigned at the Kingdom/Phylum level were removed. A cross-sectional analysis was conducted to identify the microbiome profile for each of the dietary scores computed from FFQ3. Among the 3,345 participants with 27,650 ASVs recorded, 1,610 provided dietary data on the FFQ3 and were included. The Shannon index and Pielou’s evenness index were calculated using the R package, “vegan” to determine the alpha diversity of the microbiome community (Oksanen 2017).

### Functional analysis of predicted metagenomes

Phylogenetic Investigation of Communities by Reconstruction of Unobserved States (PICRUSt2) pipeline version 2.4.1 (Douglas *et al*., 2020) was used to predict the functions of EDIH- and EDIP-enriched microbiota, following the pipeline guidelines and Metacyc dataset (Caspi *et al.*, 2020).

### Assessment of circulating biomarker data

We obtained circulating biomarker data at baseline, follow-up 1, follow-up 2, and follow-up 3 and assessed fasting insulin, glucose, and C-reactive protein (CRP; Menni *et al.*, 2013; Sas *et al*., 2017). The homeostatic model assessment of insulin resistance (HOMA-IR) was calculated from fasting insulin and fasting glucose data using the standard formula [HOMA-IR = insulin (μIU/ml) × glucose (mmol/L)/22.5 (Wallace *et al*., 2004). If one participant had multiple biomarker data in the same period, we used the mean value in this period as the final biomarker value.

### Statistical analysis

To describe participants’ characteristics, all categorical variables were presented using frequencies (%), and all continuous variables were presented using means (standard deviations) across quintiles of the dietary indices (EDIH and EDIP) at each of the three time points for dietary data collection, but using FFQ3 as the primary analysis. The Shannon index and Pielou’s evenness index were normalised via log transformation using natural logs. We estimated the percentage difference in alpha diversity per 1 standard deviation increments in the dietary index using multivariable-adjusted linear regression analyses, adjusting for all the covariates listed below. In addition, we estimated the absolute alpha diversity indices in quintiles of EDIH and EDIP via back-transformation of the log-transformed values, and the corresponding relative alpha diversity using the lowest quintile as the reference.

For microbiome profile analyses, we aggregated the microbiome data at the genus level and conducted a centred log-ratio transformation after adding one pseudo count for genera expressed in >90% of participants. We identified microbiome profiles for adherence to each dietary pattern using elastic net regression. First, we randomised the dataset into 7:3 ratio (70% for training and 30% for testing) and used an elastic net regression model within a 10-fold cross-validation framework to regress EDIH and EDIP on the 143 genera in the training dataset. Then, we applied the trained model to the testing dataset to calculate a dietary index-related microbiome profile score. The score was calculated as the weighted sum of the selected microbial genera, with weights equal to the elastic net regression coefficients. In the training dataset, we calculated the microbiome scores using the elastic net regression model with a leave-one-out validation approach to avoid over-fitting. We calculated the Spearman correlation coefficient between dietary index-related microbiome profile scores, individual genera, the dietary indices, and dietary index food components.

We conducted two levels of functional analyses: First, we used multivariable-adjusted linear regression to assess the association between dietary index-related microbiome profile scores and circulating biomarkers of insulinaemia and inflammation. Second, we used PICRUSt2 to predict the functional pathways of the metagenomes. In this analysis, we excluded pathways with more than 90% zeros and then did a probit transformation before multivariable-adjusted linear regression analyses used above. A cut-off value of 0.1 for FDR *p* value was used to screen significant pathways associated with EDIH and EDIP. For the top 10 positively and negatively associated pathways, a z-score normalised abundance in each quintile was used to generate a heatmap for data visualisation.

In addition, we used similar multivariable-adjusted linear regression analyses to assess the associations between dietary indices and biomarkers detected at different time points. To determine how closely twins share the same dietary pattern, sensitivity analyses were conducted for biomarker, microbiome alpha diversity, and the microbiome score/biomarker analysis in twin pairs only, using a mixed-effects regression model by specifying twin pairs as a random effect to account for potential within-twin pair correlation.

The following covariates were assessed and included in the multivariable-adjusted models: total energy intake (kcal/day, continuous); age at FFQ time points (years, continuous); sex (male, female); self-reported racial/ethnic group (White, Black, Asian, and other); smoking status (never, current, past smoker); number of nutrient supplements used (continuous); occupation (unemployed, retired, permanently disabled, highly paid [doctor, pharmacist, professor, lawyer]), medium-paid profession (teacher, social worker, nurse, and similar), low-paid profession (waitress, cashier, cleaner, and similar); educational levels (less than elementary school, elementary school, high school, college, and higher); postmenopausal status (menopaused, not menopaused, men); hormone replacement therapy (yes/no); mean fasted hours (hours, continuous); nonsteroidal anti-inflammatory drug use (yes/no). All analyses were additionally adjusted for BMI (BMI = weight (kg)/(height (m)^2^, continuous). For twin-pair sensitivity analyses, zygosity was additional adjusted. Data on these covariates were collected by self-administered questionnaires on demographics, medical history, and lifestyle factors at baseline and follow-up periods. All analyses were conducted using SAS® version 9.4 (SAS Institute, Cary, NC) and R.

## Results

### Participants’ characteristics

Participant characteristics in quintiles of EDIH and EDIP dietary patterns computed from FFQ3 are shown in Table 1. Participants in the highest quintile (most hyperinsulinaemic or pro-inflammatory, respectively) compared with the lowest quintile of EDIH or EDIP were predominantly non-White, had higher BMI, and lower level of education. They also reported lower intake of whole grains, higher protein, fat, and sodium intake, and lower potassium intake. Similar trends of characteristics were observed in FFQ2 (Supplementary Table 2) and FFQb (Supplementary Table 3) dietary score quintiles.

**Table 1.** Characteristics of study participants at the third dietary assessment (FFQ3) in the TwinsUK cohort

	Empirical Dietary Index for Hyperinsulinaemia (EDIH) score quintiles^a^				Empirical Dietary Inflammatory Index (EDIP) score quintiles^a^			
Characteristic^b^	Quintile 1 (*n* = 688)	Quintile 3 (*n* = 688)	Quintile 5 (*n* = 688)	*P* value^c^	Quintile 1 (*n* = 688)	Quintile 3 (*n* = 688)	Quintile 5 (*n* = 688)	*P* value^c^
Age, years	60.6 ± 13.3	59.7 ± 13.9	58.6 ± 14.4	0.004	56.8 ± 13.6	60.3 ± 13.7	60.6 ± 14.3	<.0001
Gender, (%, *n*)								
Male	12.8 (88)	8.6 (59)	9.7 (67)	0.024	15.3 (105)	8.1 (56)	6.8 (47)	<.0001
Female	87.2 (600)	91.4 (629)	90.3 (621)		84.7 (583)	91.9 (632)	93.2 (641)	
Race/ethnicity^d^, (%, *n*)								
White	89.1 (613)	90.3 (621)	86.1 (592)	0.130	87.2 (600)	90.8 (625)	85.9 (591)	0.011
Non-white	10.9 (75)	9.7 (67)	14.0 (96)		12.8 (88)	9.2 (63)	14.1 (97)	
Body mass index (BMI), kg/m^2^, (%, *n*)	24.8 ± 4.5	25.8 ± 4.7	26.9 ± 5.3	<.0001	25.0 ± 4.4	25.6 ± 4.6	26.4 ± 5.0	<.0001
Underweight (15 ≤ BMI < 18.4)	2.6 (18)	0.9 (6)	0.3 (2)	<.0001	1.7 (12)	0.9 (6)	0.9 (6)	<.0001
Normal weight (18.5 ≤ BMI < 25)	57.0 (392)	48.8 (336)	40.4 (278)		53.9 (371)	51.0 (351)	44.5 (306)	
Overweight (25 ≤ BMI < 30)	27.8 (191)	34.5 (237)	35.9 (247)		31.1 (214)	34.0 (234)	33.7 (232)	
Obese (BMI ≥30)	12.7 (87)	15.8 (109)	23.4 (161)		13.2 (91)	14.1 (97)	20.9 (144)	
NASID usage, (%, *n*)	36.9 (254)	33.0 (227)	30.4 (209)	0.490	36.5 (251)	36.3 (250)	31.0 (213)	0.106
Number of supplements used	1.0 ± 1.5	0.9 ± 1.4	0.8 ± 1.4	0.097	0.9 ± 1.4	0.9 ± 1.5	0.9 ± 1.4	0.732
Education, (%, *n*)								
Less than high school/missing	61.9 (426)	62.2 (428)	62.8 (432)	0.016	57.4 (395)	64.1 (441)	65.4 (450)	<.0001
High school	11.3 (78)	10.8 (74)	14.8 (102)		17.3 (119)	17.6 (121)	17.7 (122)	
College and higher	20.4 (140)	19.8 (136)	17.3 (119)		25.3 (174)	18.3 (126)	16.9 (116)	
Smoke status, (%, *n*)								
Current	6.1 (42)	4.8 (33)	8.3 (57)	0.101	7.9 (54)	5.4 (37)	7.4 (51)	0.036
Former	34.9 (240)	33.0 (227)	31.4 (216)		38.2 (263)	33.6 (231)	30.2 (208)	
Never	59.0 (406)	62.2 (428)	60.3 (415)		53.9 (371)	61.1 (420)	62.4 (429)	
Postmenopausal women, (%, *n*)	60.2 (414)	62.8 (432)	60.9 (419)	0.684	50.2 (345)	66.4 (457)	66.4 (457)	<.0001
Food intake^e^, servings/week								
Read meat	4.5 ± 4.1	6.6 ± 4.3	12.1 ± 8.5	<.0001	6.0 ± 5.0	6.8 ± 4.9	9.9 ± 8.5	<.0001
processed meat	2.3 ± 2.6	3.2 ± 2.4	5.8 ± 5.0	<.0001	2.9 ± 2.7	3.2 ± 2.7	5.0 ± 5.0	<.0001
Sugar-sweetened beverages	1.4 ± 3.9	1.8 ± 3.9	4.0 ± 7.6	<.0001	1.5 ± 3.8	1.4 ± 3.0	4.5 ± 8.3	<.0001
Green-leafy vegetables	3.3 ± 2.7	3.2 ± 2.2	3.8 ± 2.9	0.001	3.5 ± 3.0	3.2 ± 2.5	3.8 ± 2.8	<.0001
Refined grains	23.6 ± 15.8	19.4 ± 12.5	21.0 ± 13.4	<.0001	19.0 ± 12.4	19.6 ± 13.1	24.8 ± 16.6	<.0001
Whole grains	15.8 ± 13.8	13.7 ± 10.6	12.5 ± 10.8	<.0001	14.8 ± 13.5	13.1 ± 10.4	13.0 ± 10.5	0.001
Total fruit	25.0 ± 18.8	21.3 ± 14.5	20.0 ± 15.0	<.0001	20.7 ± 15.9	22.4 ± 16.9	24.3 ± 16.9	0.000
Wine	8.4 ± 11.8	3.5 ± 5.2	2.3 ± 3.8	<.0001	10.7 ± 12.5	3.0 ± 3.7	1.7 ± 2.9	<.0001
Tea	18.9 ± 13.7	19.1 ± 13.7	19.2 ± 13.9	0.786	19.9 ± 14.5	19.2 ± 13.6	17.3 ± 13.8	0.002
Coffee	14.2 ± 13.1	11.0 ± 10.8	9.1 ± 11.2	<.0001	15.6 ± 14.4	11.4 ± 11.4	7.8 ± 9.6	<.0001
Nutrient profile^f^								
Total energy, kcal/day	1953 ± 581	1687 ± 514	1909 ± 550	<.0001	1897 ± 563	1695 ± 541	1948 ± 572	<.0001
Total protein, g/day	38.3 ± 5.9	44.1 ± 5.6	49.5 ± 8.3	<.0001	40.2 ± 6.4	44.3 ± 6.7	47.4 ± 8.6	<.0001
Total fat, g/day	36.7 ± 6.7	37.0 ± 5.8	38.6 ± 5.9	<.0001	36.5 ± 6.5	37.5 ± 6.0	37.6 ± 6.1	<.0001
Total carbohydrate, g/day	124 ± 20	124 ± 17	116 ± 17	<.0001	118 ± 20	124 ± 17	123 ± 18	<.0001
Total fibre, g/day	10.4 ± 3.3	11.1 ± 3.3	10.5 ± 3.1	<.0001	10.2 ± 3.2	11.0 ± 3.3	11.1 ± 3.5	<.0001
Total protein, %kcal/day	33.0 ± 6.0	33.3 ± 5.2	34.8 ± 5.3	<.0001	32.8 ± 5.8	33.7 ± 5.4	33.9 ± 5.4	<.0001
Total fat, %kcal/day	15.3 ± 2.3	17.6 ± 2.3	19.8 ± 3.3	<.0001	16.1 ± 2.6	17.7 ± 2.7	19.0 ± 3.4	<.0001
Total carbohydrate, %kcal/day	50.8 ± 8.0	49.7 ± 6.7	46.6 ± 6.9	<.0001	47.3 ± 8.0	49.5 ± 6.7	49.0 ± 7.1	<.0001
Sodium, mg/day	1195 ± 295	1287 ± 316	1280 ± 285	<.0001	1165 ± 294	1261 ± 278	1300 ± 289	<.0001
Potassium, mg/day	1979 ± 395	2095 ± 374	2047 ± 374	<.0001	2028 ± 385	2110 ± 389	2035 ± 389	0.000
Calcium, mg/day	590 ± 174	558 ± 152	487 ± 134	<.0001	556 ± 173	564 ± 156	514 ± 148	<.0001
Magnesium, mg/day	181 ± 35	182 ± 31	172 ± 30	<.0001	181 ± 33	183 ± 33	174 ± 33	<.0001
Zinc, mg/day	5.0 ± 0.8	5.5 ± 0.8	5.9 ± 1.1	<.0001	5.1 ± 0.8	5.5 ± 0.9	5.7 ± 1.0	<.0001

a Dietary indices were adjusted for total energy intake using the residual method.

b Values presented are means ± SD for continuous variables and percentages for categorical variables.

c *P* value for differences of participant characteristics across quintiles. *P* values were calculated using chi square test for categorical variables and ANOVA for continuous variables.

d In TwinsUK, race/ethnicity was self-identified. Non-white ethnicity group included Black, Asian, mixed and others.

e The food group variables (servings/day) in TwinsUK were as follows: processed meat (Bacon, corned beef, spam, luncheon meats, ham, and sausages); red meat (roast, steak, mince, stew or casserole beef, savoury pies, for example, meat pie, pork pie, pasties, steak and kidney pie, sausage roll, roast, chops, stew or slices pork, beefburgers, meat soups, and roast, chops or stew lamb); sugar-sweetened beverages all regular (not diet) soft drinks and sweetened fruit drinks; wine (white wine); coffee or tea (all types); green leafy vegetables (green salad, lettuce, cucumber, celery, spinach, broccoli, spring green, kale, and watercress).

f Nutrient values are nutrient densities, presented per 1000 kcal of total energy intake.

### Dietary indices in relation to microbiota alpha diversity

In cross-sectional analyses using FFQ3 dietary data, higher EDIH and EDIP, reflecting more hyperinsulinaemic or more pro-inflammatory dietary patterns, were associated with lower microbiota alpha diversity, as the Shannon index decreased 3.2% and 2.3% comparing the highest quintiles to the lowest quintiles for EDIH and EDIP, respectively. Similarly, Pielou’s evenness index also decreased 2.1% and 1.4% for the same comparisons. For both dietary patterns scores, the associations were slightly attenuated with additional adjustment for BMI (Table 2). Similar results were found for longitudinal assessments of alpha diversity with the EDIH and EDIP scores calculated from FFQ2 and FFQb (Supplementary Table 4).

**Table 2.** Multivariable-adjusted absolute and relative value (95% CI) of α-diversity in quintiles of the dietary indices in FFQ3

Statistical model	Alpha diversity index		Quintile 1	Quintile 3	Quintile 5	Percentage difference per SD	FDR *p*-value^c^
Empirical Dietary Index for Hyperinsulinaemia (EDIH) score							
MV^a^	Shannon diversity	Absolute value	3.60 (3.40, 3.80)	3.55 (3.35, 3.75)	3.49 (3.30, 3.69)	**−1.0 (−1.7, −0.3)**	**0.037**
		Relative value	1 (ref)	−1.4 (−4.1, 1.3)	−3.2 (−5.9, −0.4)		
	Pielou’s evenness	Absolute value	0.68 (0.65, 0.71)	0.68 (0.65, 0.71)	0.67 (0.64, 0.70)	**−0.6 (−1.2, −0.1)**	**0.044**
		Relative value	1 (ref)	−1.0 (−3.1, 1.1)	−2.1 (−4.2, 0.04)		
MV + BMI^b^	Shannon diversity	Absolute value	3.60 (3.41, 3.81)	3.57 (3.37, 3.78)	3.52 (3.33, 3.72)	−0.8 (−1.5, −0.04)	0.061
		Relative value	1 (ref)	−0.9 (−3.6, 1.8)	−2.4 (−5.2, 0.4)		
	Pielou’s evenness	Absolute value	0.68 (0.65, 0.71)	0.68 (0.65, 0.71)	0.67 (0.64, 0.70)	−0.6 (−1.1, −0.01)	0.061
		Relative value	1 (ref)	−0.8 (−3.0, 1.3)	−1.8 (−4.0, 0.3)		
Empirical Dietary Inflammatory Pattern (EDIP)							
MV^a^	Shannon diversity	Absolute value	3.59 (3.39, 3.79)	3.58 (3.39, 3.79)	3.50 (3.31, 3.70)	**−0.8 (−1.5, −0.1)**	**0.048**
		Relative value	1 (ref)	−0.1 (−2.8, 2.7)	−2.3 (−5.1, 0.4)		
	Pielou’s evenness	Absolute value	0.68 (0.65, 0.71)	0.68 (0.65, 0.71)	0.67 (0.64, 0.70)	**−0.6 (−1.2, −0.1)**	**0.045**
		Relative value	1 (ref)	−0.3 (−2.4, 1.8)	−1.4 (−3.6, 0.7)		
MV + BMI^b^	Shannon diversity	Absolute value	3.60 (3.40, 3.80)	3.60 (3.40, 3.81)	3.53 (3.34, 3.73)	−0.7 (−1.4, 0.05)	0.071
		Relative value	1 (ref)	0.2 (−2.6, 2.9)	−1.9 (−4.7, 0.8)		
	Pielou’s evenness	Absolute value	0.68 (0.65, 0.71)	0.68 (0.65, 0.71)	0.67 (0.64, 0.70)	−0.6 (−1.1, −0.04)	0.059
		Relative value	1 (ref)	−0.2 (−2.3, 1.9)	−1.3 (−3.4, 0.8)		

a Values are beta-coefficients from linear regression models, adjusted for total energy intake, BMI, age, race, smoking status, supplement use, occupation, education, postmenopausal status, hormone replacement therapy, and nonsteroidal anti-inflammatory drug use.

b BMI was additional adjusted.

c The bolded numbers represent statistically significant findings (i.e., FDR *p* value <0.05).

### Dietary indices-related microbiome profile scores

Dietary index-related microbiome scores at the genus level were developed using FFQ3 data. The elastic net regression retained 32 and 15 genera to compute the EDIH- and EDIP-related microbiome scores, respectively. For EDIH, *Caproiciproducens* (β = −0.034)*, Intestinimonas* (β = −0.025), and *Ruminococcaceae UCG-008* (β = −0.024) showed the largest inverse associations (i.e., low insulinaemic), whereas *Adlercreutzia* (β = 0.057), *Negativibacillus* (β = 0.023), and *Turicibacter* (β = 0.019) showed the largest positive associations (i.e., hyperinsulinaemic), (Supplementary Table 5). For EDIP, *Ruminococcaceae_UCG-008* (β = −0.020)*, Lachnospiraceae_UCG-008* (β = −0.015), and *Defluviitaleaceae_UCG-011* (β = −0.012) showed the largest inverse associations (low inflammatory), while *Streptococcus* (β = 0.010)*, Eisenbergiella* (β = 0.008), and *Negativibacillus* (β = 0.008) showed the largest positive associations (Supplementary Table 6). Six genera were associated with both EDIH and EDIP, four inversely (*Ruminococcaceae_UCG-008, Lachnospiraceae_UCG-008, Defluviitaleaceae_UCG-011, Anaeroplasma*), and two positively (*Negativibacillus, Streptococcus*).

**Figure 1.**
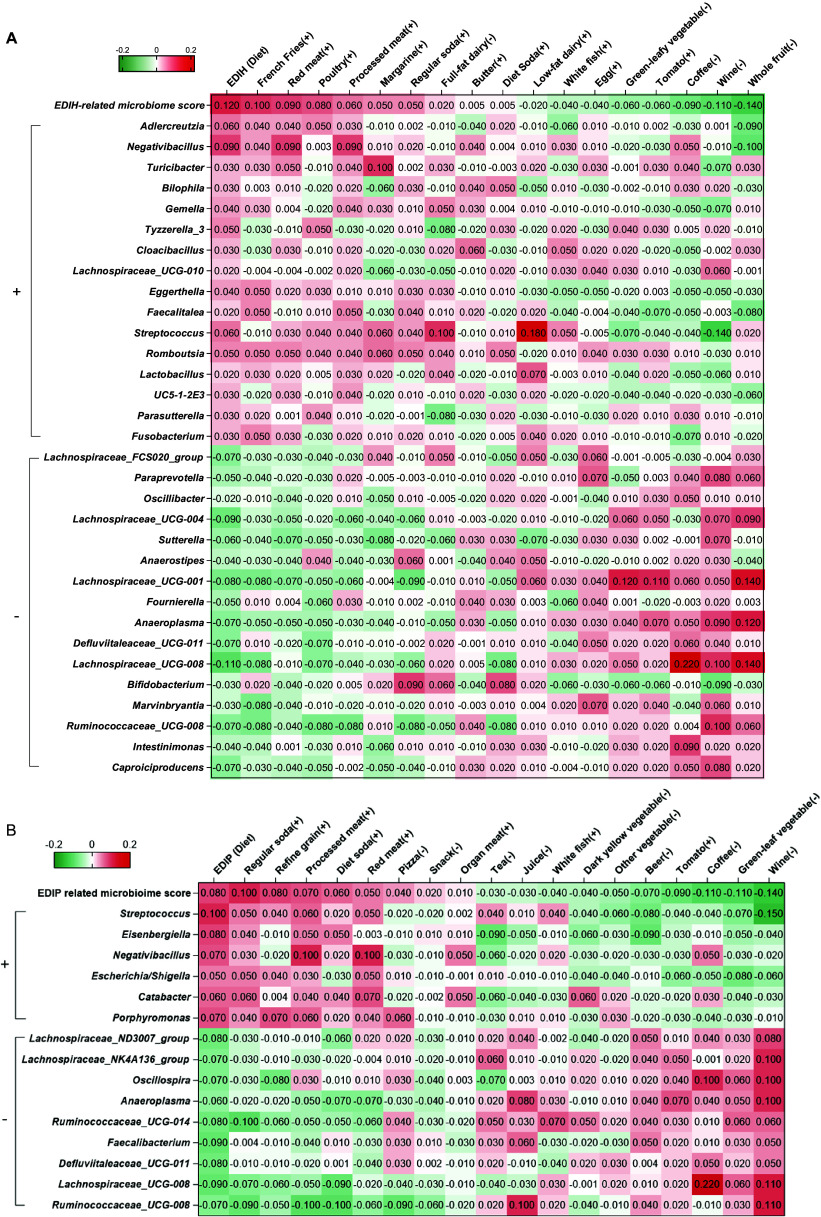
Heatmap showing Spearman correlations between (a) 32 genera comprising the EDIH-related microbiome profile score, the EDIH dietary score and its food group components and (b) 15 genera comprising the EDIP-related microbiome profile score, the EDIP dietary score, and its food group components. The ± symbol after the food component name represents the food components positively or negatively associated with the dietary index. The ± symbol before the individual genera represents the genera positively or negatively associated with the microbiome score.

The microbiome profile scores showed similar correlations (as the dietary scores) with dietary score food components. That is, microbiome profile scores were positively correlated with food components contributing to higher dietary scores, while inversely correlated with food components contributing to lower dietary scores. Single genera showed the same positive or inverse correlations with microbiome profile and dietary scores. For the microbiota associated with both EDIH and EDIP, the *Lachnospiraceae* were positively associated with green leafy vegetables, coffee, wine, and whole fruit, while *Negativibaccilus, streptococcus*, and *Adlercreutzia* seem to positively associate with French fries, red/processed meat, poultry, regular sodas, and diet sodas (Figure 1, Supplementary Tables 5–7).

### Microbiome functional analysis

Higher EDIH- and EDIP-related microbiome profile scores, reflecting microbes associated with a more hyperinsulinaemic or more pro-inflammatory microbiome profile, were significantly associated with higher concentration of insulin, glucose, and HOMA-IR in cross-sectional analyses at follow-up 3. However, the results were attenuated for glucose when additionally adjusted for BMI (Table 3). In addition, the two microbiome scores were significantly associated with higher concentration of insulin and HOMA-IR in longitudinal analysis at follow-up 2. However, we did not observe significant associations with baseline dietary scores assessed on average 12 year earlier.

**Table 3.** Multivariable-adjusted beta coefficient (95% CI) for the associations of the dietary index-related microbiome profile scores with circulating biomarkers of insulinaemia and inflammation

			EDIH microbiome profile score		EDIP microbiome profile score	
	Biomarkers		β coefficient	FDR-*p*	β coefficient	FDR-*p*
FFQ3	Fasting insulin, μU/mL (*n* = 1501)^a^ ^,^^b^	MV	0.7 (0.5, 0.9)	**<0.001**	1.4 (1.1, 1.8)	**<0.001**
		MV + BMI	0.3 (0.1, 0.4)	**0.002**	0.6 (0.3, 0.9)	**<0.001**
	Fasting glucose, mg/dL (*n* = 1541)	MV	0.04 (0.01, 0.1)	**0.009**	0.1 (0.02, 0.1)	**0.015**
		MV + BMI	0.02 (−0.02, 0.1)	0.332	0.02 (−0.04, 0.1)	0.492
	HOMA-IR^c^ (*n* = 1501)	MV	0.7 (0.5, 0.9)	**<0.001**	1.5 (1.1, 1.9)	**<0.001**
		MV + BMI	0.3 (0.1, 0.5)	**0.002**	0.6 (0.3, 1.0)	**0.001**
FFQ2	Fasting insulin, μU/mL (*n* = 762)	MV	0.6 (0.3, 0.9)	**<0.001**	1.2 (0.7, 1.7)	**<0.001**
		MV + BMI	0.4 (0.2, 0.7)	**<0.001**	0.6 (0.2, 1.1)	**0.010**
	Fasting glucose, mg/dL (*n* = 804)	MV	0.04 (0.001, 0.1)	**0.057**	0.1 (−0.03, 0.1)	0.206
		MV + BMI	0.03 (−0.01, 0.1)	0.170	0.02 (−0.1, 0.1)	0.685
	HOMA-IR (*n* = 761)	MV	0.7 (0.4, 0.9)	**<0.001**	1.2 (0.7, 1.8)	**<0.001**
		MV + BMI	0.5 (0.2, 0.7)	**<0.001**	0.7 (0.2, 1.2)	**0.012**
FFQb	Fasting insulin, μU/mL (*n* = 377)	MV	0.2 (−0.2, 0.7)	0.333	0.8 (−0.2, 1.7)	0.122
		MV + BMI	0.2 (−0.3, 0.6)	0.529	0.5 (−0.5, 1.5)	0.306
	Fasting glucose, mg/dL (*n* = 638)	MV	−0.30 (−0.1, 0.02)	0.179	0.03 (−0.1, 0.1)	0.521
		MV + BMI	−0.04 (−0.1, 0.01)	0.083	−0.002 (−0.1, 0.1)	0.963
	HOMA-IR^c^ (*n* = 377)	MV	0.2 (−0.3, 0.7)	0.450	0.8 (−0.2, 1.8)	0.125
		MV + BMI	0.1 (−0.4, 0.6)	0.703	0.5 (−0.5, 1.5)	0.336

a Values are beta coefficients from the multivariable-adjusted linear regression and the bolded numbers represent statistically significant findings (i.e., FDR *p* value <0.05).

b Values were adjusted for total energy intake, age, sex, race, smoking status, supplement use, occupation, education, postmenopausal status, nonsteroidal anti-inflammatory drug use, and hormone replacement therapy.

c Abbreviations: HOMA-IR, Homeostatic model assessment of insulin resistance.

**Figure 2.**
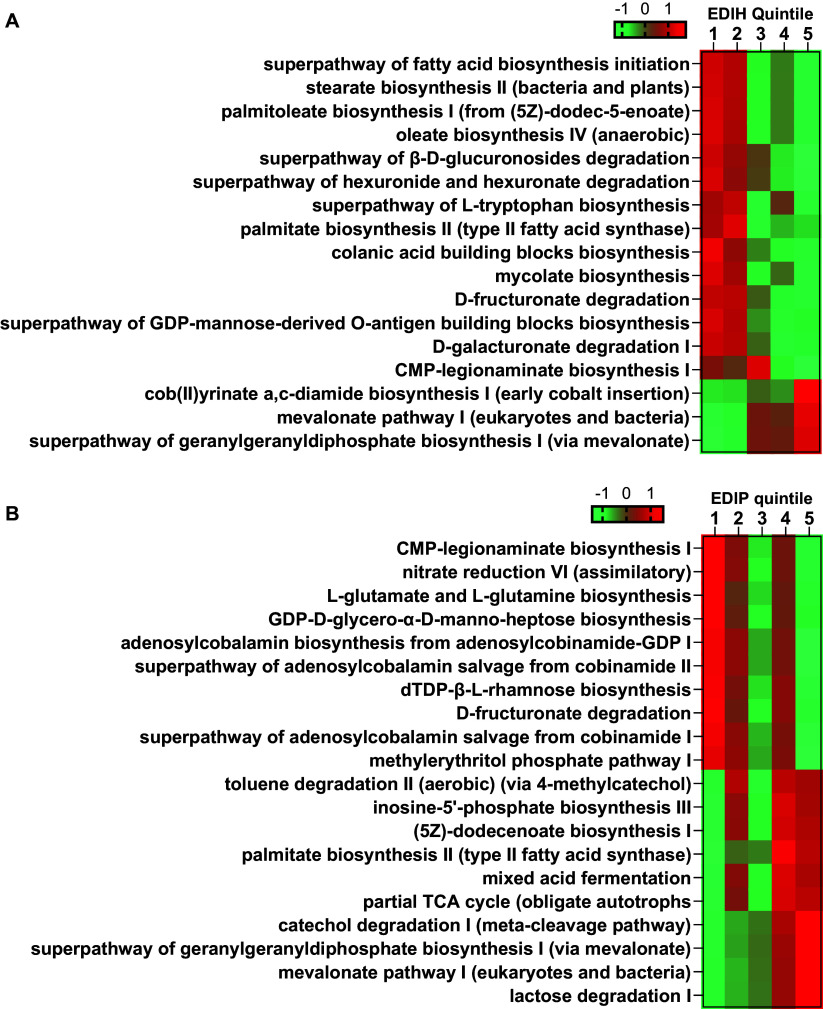
Heatmap showing the z-score standard expression of (a) 17 significant pathways screened out from PICRUST and multivariable-adjusted linear regression across the quintiles of EDIH and (b) top 10 significantly positive and negative pathways screened out from PICRUST2 and multivariable-adjusted linear regression across the quintiles of EDIP.

In the predicted pathways analysis, multivariable-adjusted linear regression identified 14 pathways inversely associated with EDIH and 3 pathways positively associated. Among the 14 inverse pathways, 4 of them, involved in the biosynthesis of fatty acids (fatty acid, stearate, palmitoleate, oleate), were still significantly associated after additionally adjusting for BMI. The mevalonate pathway is the major positively associated pathways for EDIH. Multivariable-adjusted linear regression identified 33 pathways inversely associated with EDIP and 121 pathways positively associated. Higher EDIP score was associated with down-regulation of pathway abundances for the biosynthesis of nucleotide sugars (e.g., CMP-legionaminate, GDP-d-glycero-α-d-manno-heptose and dTDP-l-rhamnose), and amino acid (L-glutamate and L-glutamine), nitrogen compound metabolism, adenosylcobalamin salvage, D-fructuronate degradation; and up-regulation of pathway abundances for the biosynthesis of inosine-5′-phosphate, fatty acid ((5Z)-dodecenoate, palmitoleate), and geranylgeranyl diphosphate (GGPP), degradation of aromatic compounds (e.g., catechol and toluene) and lactose, mixed acid fermentation, TCA cycle, and mevalonate pathway (Figure 2).

### Dietary index scores and circulating biomarker concentrations

In the construct validation sub-study, we found that higher EDIH and EDIP scores were significantly associated with higher concentrations of insulin, HOMA-IR, and CRP but not glucose in TwinsUK in cross-sectional analyses. Longitudinal analyses confirmed that EDIH and EDIP score assessed multiple years earlier were still associated with future unfavourable plasma biomarker profiles (Supplementary Figure 2).

### Sensitivity analysis among twin pairs

We had 1,520; 1,039; and 1,075 twin pairs at FFQb, FFQ2, and FFQ3 twin-pair analyses. The mixed-effects regression results showed very similar results for alpha diversity and microbiome scores when compared with the main analysis results (Supplementary Tables 8 and 9). Paired t tests did not show any significant differences in dietary scores, alpha diversity scores, and microbiome profile scores between twin pairs (data not shown).

## Discussion

Dietary patterns with a high potential to contribute to insulin hypersecretion and chronic systemic inflammation, based on higher EDIH and EDIP scores, have been associated with multiple metabolic diseases in previous studies (Wang *et al*., 2023). To explore the potential mechanisms that may underlie such associations, we identified microbiome diversity changes and differences in the abundance of specific microbes linked to hyperinsulinaemic and pro-inflammatory dietary patterns for the first time. Specifically, more hyperinsulinaemic and pro-inflammatory dietary patterns were associated with lower faecal microbial diversity and the abundances of specific microbes, essential biosynthetic and degradation processes in metabolic pathways, providing insights on which microbes may be depleted and/or enriched in a dietary pattern intervention to lower the insulinaemic or inflammatory activity of the diet. The related microbiome profile scores of the dietary patterns were also predictive of the plasma biomarker constructs of EDIH and EDIP. In the construct validation study, we determined that more hyperinsulinaemic or pro-inflammatory dietary patterns (higher EDIH and EDIP) were significantly associated with higher concentrations of insulin, HOMA-IR, and CRP, but not glucose. These findings from the three cross-sectional analyses were confirmed in the four longitudinal analyses, suggesting that dietary patterns assessed multiple years earlier, may impact future plasma biomarker profiles.

A major objective of the dietary patterns approach to nutrition research is to examine their impact on health outcomes; however, one limitation of the prevailing dietary patterns is that most are not designed to optimise disease prediction, and for this reason, metabolic dietary patterns, including the EDIH and EDIP, have been proposed (Shi *et al.*, 2021, Wang *et al*., 2023). This approach to dietary patterns is based on the premise that a dietary pattern predictive of a biological marker in a disease pathway (e.g., hyperinsulinaemia, lipids, chronic systemic inflammation) may be more predictive of disease outcomes if the pathway is a strong determinant of the disease. Indeed, previous studies found that both EDIH and EDIP were more predictive of: (i) type 2 diabetes risk than the glycaemic index and glycaemic load (Jin *et al*., 2021; Lee *et al*., 2020), (ii) colorectal cancer risk (Wang *et al*., 2022), and risk of major chronic diseases (Wang *et al*., 2023), than existing dietary pattern indices such as the Healthy Eating Index (HEI), Dietary Approaches to Stop Hypertension diet, and so forth.

Multiple previous studies have investigated the associations of several indices of dietary quality and the gut microbiome, and generally found that higher dietary quality is associated with higher microbiota diversity, which can improve immune function and digestion and reduce the risk of metabolic and inflammatory diseases. A study in the Multi-Ethnic Cohort found that higher dietary quality based on four different dietary quality indices was associated with higher alpha diversity and specific taxa (Maskarinec *et al*., 2019). Moreover, diet quality scores are associated with higher abundances of specific microbes that are potentially beneficial for health improvement (Bowyer *et al*., 2018; Maskarinec *et al*., 2019). Our results align with previous studies that have found fibre-fermenting bacteria, including *Faecalibacterium, Lachnospira*, and *Ruminococcus*, to be associated with higher dietary quality. These are among three of the six genera or family (for *Faecalibacterium and Negativibacillus*) that were inversely associated with higher EDIH or EDIP in the current study. Although we did not analyse other dietary patterns in this study, a previous study among 2,070 individuals in the TwinsUK cohort showed that two commonly used dietary quality indices – the HEI-2010 and the Mediterranean Dietary Score – were associated with higher alpha diversity (Bowyer *et al*., 2018). The EDIH and EDIP assess dietary quality based on the insulinaemic or inflammatory potential of the diet, and lower EDIH/EDIP scores correlate positively with the conventional indices of dietary quality (Wang *et al*., 2023; Wang *et al*., 2022). Although EDIH, EDIP, and HEI are all food-based dietary patterns, EDIH and EDIP are specifically designed based on biological mechanisms, whereas HEI is aimed at evaluating adherence to the Dietary Guidelines for Americans.

We created microbiome profile scores to better reflect the role of the dietary pattern in the microbiota composition and further demonstrate function by relating these microbiome profile scores with circulating markers of inflammation and insulin response, or through predicting the pathways of metagenomes. Based on the current study findings, to improve metabolic and overall health, a dietary intervention to reduce inflammatory or insulinaemic activity of the diet may therefore tip the overall balance of the gut microbial composition to greater levels of *Lachnospiraceae, Faecalibacterium, Oscillospira, Ruminococcaceae_UCG-014, Marvinbryantia, Intestinimonas*, and *Anaerostipes*, genera, and reduced levels of *Porphyromonas, Eisenbergiella, Escherichia/Shigella, Adlercreutzia, Eggerthella, Fusobacterium*, and *Bilophila.* Functional analysis revealed that EDIH and EDIP might regulate essential biosynthetic and biodegradation processes involved in nutrient assimilation, energy production, and the synthesis of critical biomolecules necessary for cellular structure and function. Several lipid metabolism pathways were found in the functional analysis. Higher EDIH was associated with reduced biosynthesis of monounsaturated fatty acid (MUFA), oleic acid, and palmitic acid. We also observed that higher EDIP was associated with increased palmitate biosynthesis. The increased palmitic acid may attenuate the insulin signalling pathway and promote insulin resistance through decreasing the function of the endoplasmic reticulum (ER) and mitochondria. In addition, palmitic acid can activate pro-inflammatory pathways through Toll-like receptor 4 pathways. Another MUFA, oleic acid, has the potential to attenuate these unhealthy functions caused by increased palmitic acid (Palomer *et al.*, 2018). An increased mevalonate pathway was associated with both EDIH and EDIP. The mevalonate pathway was upregulated by both hyperinsulinaemic and pro-inflammatory dietary patterns and produces essential regulators of cellular metabolism, such as lipoproteins, dolichol, ubiquinone, and cholesterol-derived products (Guerra *et al.*, 2021). The dysregulation of mevalonate was associated with multiple metabolic diseases, including cardiovascular disease, inflammatory bowel disease, and cancers (Guerra *et al.*, 2021, Pereira *et al*., 2022). Mevalonate is essential for GGPP biosynthesis (Guo *et al*., 2022). The inhibition of GGPP pathways showed anticancer effects in several cancers.

Our study has several strengths. We note that the EDIH and EDIP scores were developed, validated, and applied in several large cohorts, all in the United States, and this is the first time the dietary scores have been applied in a non-US population, using a robust methodology for standardizing food serving size definitions. We took advantage of the comprehensive data assessments in TwinsUK and implemented a robust study design comprised of multiple cross-sectional and longitudinal studies, yielding highly concordant results. The elastic net regression with 10-fold cross validation or leave-one-out validation approach we used here can effectively deal with highly correlated variables and, at the same time, maintain the quality of model selection compared with multiple linear regressions for each microbe as the outcome variable. However, our study is not without limitations, which include the potential for measurement error in the diet and lifestyle variables. The potential for confounding by unmeasured variables or residual confounding by inadequately measured variables may not be completely removed even as we adjusted for several potential confounding factors. Microbiome studies are generally limited by the use of relative abundances rather than absolute concentrations (Goodrich *et al*., 2016). Multiple software (bioinformatic pipelines) and algorithms are used in different studies with different limitations and biases (Nearing *et al.*, 2022; Prodan *et al.*, 2020). We regressed the microbiome profile score on the aggregated genus level but not on the species, which may miss some signals in the microbial community. Therefore, metagenomic and metatranscriptomic data may be needed in future studies. Twin studies offer valuable insights into environment–genetic interactions within microbiomes (Goodrich *et al*., 2016). However, in our study, we primarily focused on individual-level microbiome analysis, although we briefly explored twin-pair analysis in the smaller sample size. Future research, integrating microbiome and genetic data, is essential to further elucidate these interactions and their impacts on microbiome composition and variation.

## Conclusion

Dietary patterns that exert metabolic effects on insulin and inflammation may influence disease risk and prognosis by modulating gut microbial composition and function via multiple biosynthetic and biodegradation pathways, providing insights on which microbes may be depleted and/or enriched in a dietary pattern intervention to reduce the insulinaemic or inflammatory activity of the diet.

## Supporting information

Shi et al. supplementary material 1 Shi et al. supplementary material

Shi et al. supplementary material 2 Shi et al. supplementary material

Shi et al. supplementary material 3 Shi et al. supplementary material

## Data Availability

The data used in this study are held by the Department of Twin Research at King’s College London. The data can be released to bona fide researchers using our normal procedures, which are overseen by the Wellcome Trust and its guidelines, as part of our core funding (https://twinsuk.ac.uk/resources-for-researchers/access-our-data/).

## Abbreviations

EDIH: Empirical Dietary Index for Hyperinsulinaemia
EDIP: Empirical Dietary Inflammatory Pattern (EDIP)
FFQ: food frequency questionnaires
HOMA-IR: homeostasis model assessment of insulin resistance
CRP: C-reactive protein
SD: standard deviation
NDSR: Nutrition Data System for Research
EPIC: European Prospective Investigation into Cancer and Nutrition
FDA: Food and Drug Administration
ASVs: amplicon sequence variants
FDR: false discovery rate
BMI: body mass index
SCFA: Short-chain fatty acids

## Supplementary material

The supplementary material for this article can be found at http://doi.org/10.1017/gmb.2024.14.
