# Supplementary material for: Pro-inflammatory and hyperinsulinaemic dietary patterns are associated with specific gut microbiome profiles: a TwinsUK cohort study
Source: Gut Microbiome (Camb). 2024 Dec 5;5:e12. doi: 10.1017/gmb.2024.14 (PMC11658949; doi:10.1017/gmb.2024.14)
Supplement: Shi et al. supplementary material 1 — Shi et al. supplementary material [file S2632289724000148sup001.pdf]

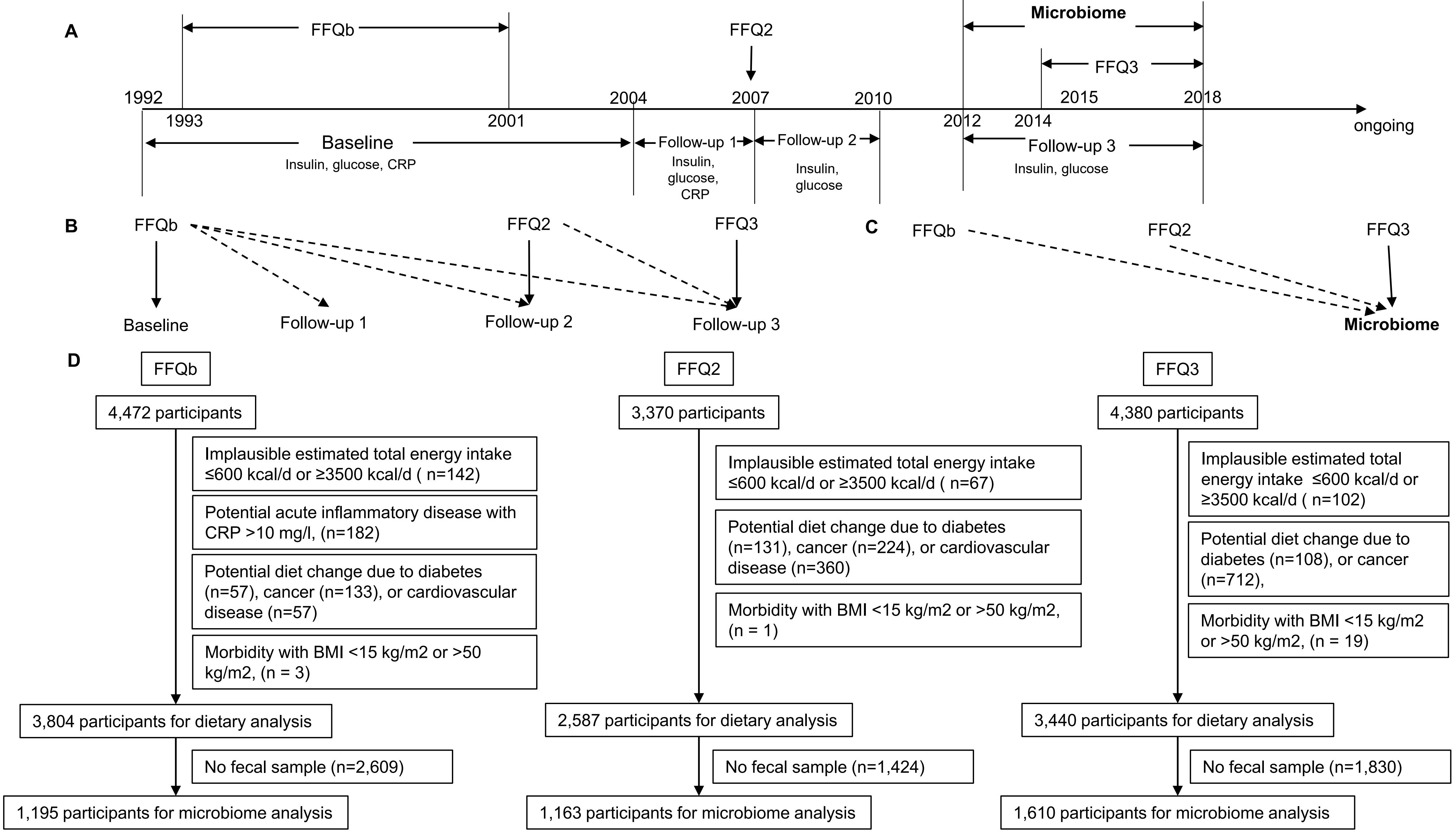

Supplementary Figure 1. Study design of the TwinsUK. A. Timeline of follow-up data collection in TwinsUK and the cross-sectional and prospective analyses design in this study. A, timeline for diet and biomarker assessment; B, biomarker statistical analyses; C, microbiome statistical analyses. Dotted arrows indicate prospective analyses, whereas the solid arrows indicate cross-sectional analyses. D. Flow chart of participants in the TwinsUK cohort for analysis.
