# Supplementary material for: Pro-inflammatory and hyperinsulinaemic dietary patterns are associated with specific gut microbiome profiles: a TwinsUK cohort study
Source: Gut Microbiome (Camb). 2024 Dec 5;5:e12. doi: 10.1017/gmb.2024.14 (PMC11658949; doi:10.1017/gmb.2024.14)
Supplement: Shi et al. supplementary material 2 — Shi et al. supplementary material [file S2632289724000148sup002.pdf]

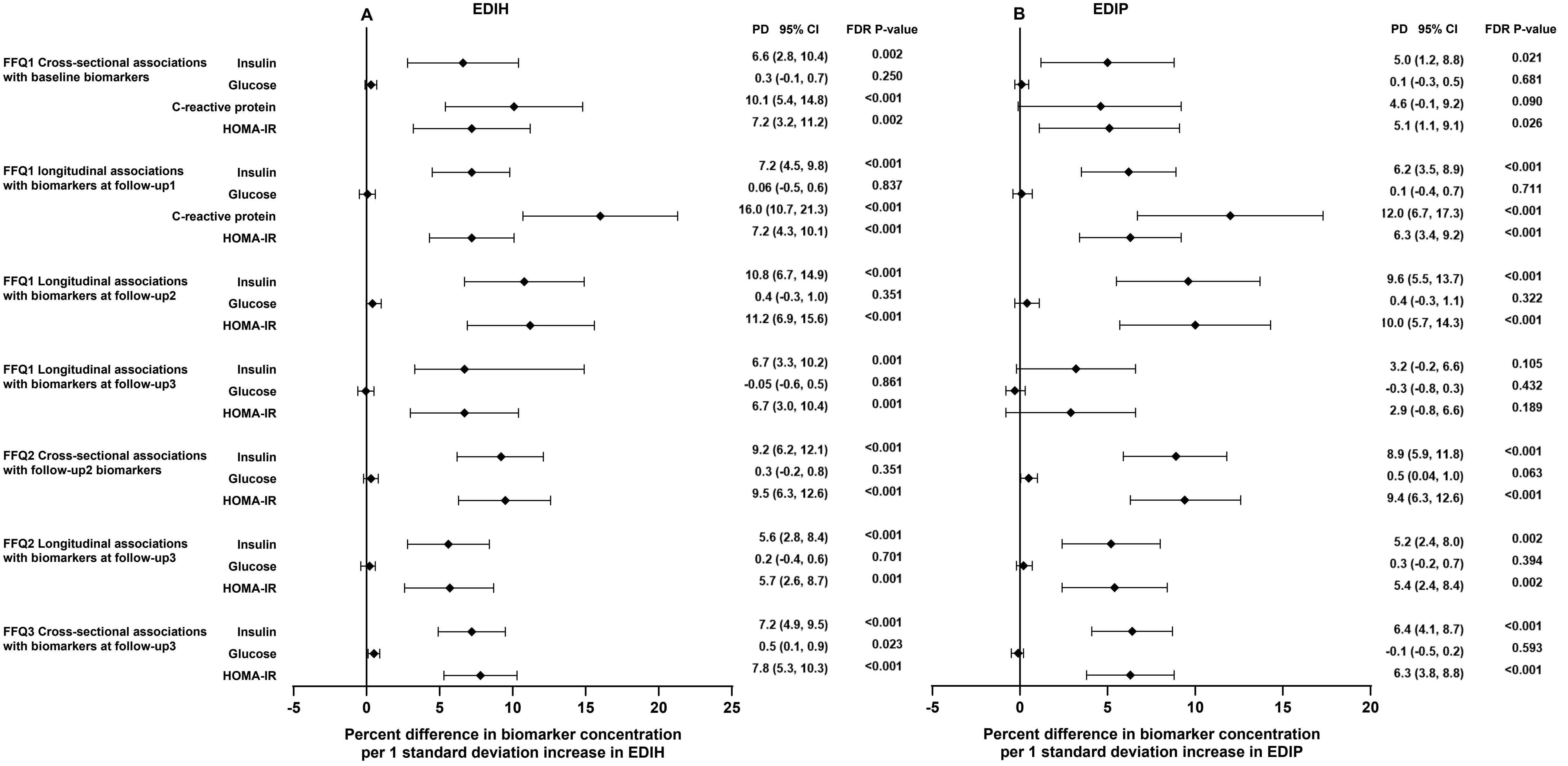


**Supplementary Figure 2 Percentage difference (PD - beta coefficients) (95% confidence intervals) in biomarker concentrations per 1 standard deviation increment in EDIH (A) and EDIP (B) in TwinsUK. Biomarkers were log-transformed using natural logs. Values were obtained via multivariable-adjusted linear regression models adjusted for the following variables: total energy intake, age, sex, race, smoking status, nutrient supplement use, physical activity, occupation, education, postmenopausal status, and hormone replacement therapy. Fasting hours, hypertension and high cholesterol status were additionally adjusted in FFQ1. Nonsteroidal anti-inflammatory drug use was additionally adjusted in FFQ3. Physical activity was not available in FFQ3.**
